# Antioxidant and Hypoglycemic Effects of Acidic-Extractable Polysaccharides from *Cordyceps militaris* on Type 2 Diabetes Mice

**DOI:** 10.1155/2018/9150807

**Published:** 2018-11-25

**Authors:** Huajie Zhao, Qiangqiang Lai, Jianjun Zhang, Chunyan Huang, Le Jia

**Affiliations:** ^1^Institute of Agricultural Resources and Environment, Shandong Academy of Agricultural Science, Key Laboratory of Wastes Matrix Utilization, Ministry of Agriculture, Jinan 250100, China; ^2^College of Life Science, Shandong Agricultural University, Tai'an 271018, China

## Abstract

The present work was performed to evaluate the effect of acidic-extractable polysaccharides (AE-PS) from fruit bodies of *Cordyceps militaris* on type 2 diabetes mellitus (T2DM) and its structural characteristics. The T2DM mice induced by high-fat diet (HFD) and streptozotocin (STZ) were administered with 100 and 400 mg/kg AE-PS for 4 weeks. Our work proved that AE-PS decreased the levels of serum lipid, lipid peroxidation, and blood glucose; improved glucose and insulin resistance; enhanced antioxidant enzyme activities; and attenuated the injuries of the liver, kidney, and pancreas in T2DM mice. These results might offer references for the exploitation of AE-PS as functional foods or natural drug source for preventing and treating HFD- and STZ-induced T2DM. Moreover, gas chromatography (GC) results revealed that AE-PS was heterogeneous and composed of fucose, ribose, arabinose, xylose, mannose, galactose, and glucose with mass percentages of 1.23%, 0.57%, 0.29%, 2.12%, 2.73%, 4.66%, and 88.4%, respectively. Fourier-transform infrared (FTIR) and nuclear magnetic resonance (NMR) analysis indicated that AE-PS was a pyran-type polysaccharide with *α*- and *β*-configurations.

## 1. Introduction

Diabetes mellitus (DM), a serious chronic endocrine metabolic disease, has caused 8.5% global adults to suffer painful torture in 2014, and this growing trend increased to 366 million or more by the year 2030 based on International Diabetes Federation reports and will bring a great economic burden to the society [1]. DM is divided into three types including type 1 diabetes mellitus, type 2 diabetes mellitus (T2DM), and gestational diabetes mellitus according to clinical manifestation [2]. T2DM, namely non-insulin-dependent DM or adult-onset diabetes, is characterized by hyperlipidemia and hyperglycemia resulting from the insulin resistance in peripheral tissues or impaired insulin synthesis in the pancreas, and it has accounted for over 90% of diabetes patients [2–4]. Nowadays, lifestyle and dietary factors such as low-cost, high-fat, and high-calorie diet consumptions have caused the number of T2DM patients growing, particularly obesity [5, 6]. Meantime, some researchers have reported that the generation of reactive oxygen species and consequent oxidative damages particularly in the liver, kidney, and pancreas can cause the symptom of high blood sugar level [7]. Hence, the supplement of antioxidants is very helpful for attenuating the symptom of high blood sugar level, thereby preventing and treating the DM and its complications. Clinically, some drugs such as thiazolidinediones and *α*-glucosidase inhibitors are proposed as T2DM treatment. However, the application of these synthetic drugs is restricted by their adverse effects such as gastrointestinal and cardiovascular events [8, 9]. Therefore, more and more researchers have attached importance to exploit and manufacture natural antidiabetic substances.

*Cordyceps militaris*, widely applied to treat diverse diseases as a traditional medicine in China, has received more and more attentions in recent years. Due to the existence of many biological active materials such as carbohydrates, protein, fat, fiber, trace elements, ash, and cordycepin, *C. militaris* possesses many beneficial bioactivities including anti-inflammatory, antihyperlipidemic, enhancing insulin resistance and insulin secretion, antibacterial, antitumor, antioxidant, immune modulating, and antivirus [10–14]. Meantime, it can also ameliorate reproductive function and treat cyclophosphamide-induced reproductive dysfunction in mice [15]. Mushroom polysaccharides, isolated from fruit body, mycelium, and fermentation broth, have been used as a source of therapeutic agents for treating hyperlipidemia, hyperglycemia, hepatic injury, and so on [16, 17].

Accumulated literatures have reported that polysaccharides, which were the major bioactive substances of *C. militaris*, have become a scientific research hotspot due to the potential biological activities. Liu et al. have reported polysaccharides from their own immunomodulatory and antioxidant activities [18]. Bohn and BeMiller [19] and Park et al. [20] have shown that *C. militaris* polysaccharides possessed anti-inflammatory and antitumor activities. Furthermore, many reports revealed that the obtained methods of *C. militaris* polysaccharides are mainly hot-water and ultrasonic extractions [18, 21]. However, few reports have been published about acidic-extractable polysaccharides (AE-PS) from the fruit bodies of *C. militaris*; its biological activities in T2DM mice containing antioxidant, hypoglycemic, and protective effects on the liver and kidney; and its characterizations. Hence, the present work aimed at investigating the hypoglycemic, antioxidant, and protective effects on the liver and kidney of AE-PS from *C. militaris* in high-fat diet- (HFD-) and streptozotocin- (STZ-) induced T2DM mice. In addition, its structure features were also processed.

## 2. Materials and Methods

### 2.1. Materials and Chemicals

The fruiting body of *C. militaris* was obtained from Beijing Engineering Research Center for Edible Mushroom (Beijing, China). The diagnostic kits for analyzing superoxide dismutase (SOD), glutathione peroxide (GSH-Px), catalase (CAT), and malondialdehyde (MDA) were purchased from Nanjing Jiancheng Bioengineering Institute (Nanjing, China). 1,1-Diphenyl-2-picrylhydrazyl (DPPH), STZ, and monosaccharide standard samples (arabinose, galactose, glucose, fucose, mannose, rhamnose, ribose, and xylose) were provided by Sigma Chemicals Co. Ltd. (St. Louis, USA). All other reagents used in this experiment were analytical grade and purchased by local chemical suppliers. Kunming mice (male, 18–22 g) were purchased from Taibang Biological Products Co. Ltd. (Tai'an, China).

### 2.2. Preparation of AE-PS

The AE-PS was prepared using the method reported by Lin et al. [22]. Briefly, the dried powder of *C. militaris* was mixed with the proper volumes of hydrochloric acid (0.5 M, 1 : 10, *w*/*v*) at 80°C for 6 h. The supernatant was collected by centrifugation (3000 rpm, 10 min) and precipitated by 3 volumes of ethanol (95%, *v*/*v*) at 4°C overnight. The obtained precipitate was deproteinated according to the Sevage method [23], dialyzed with deionized water, and lyophilized to yield AE-PS, which was used for a further work.

### 2.3. In Vitro Antioxidant Analysis

The reducing power was determined using a previously reported method [24]. In brief, AE-PS (1 mL, 0–400 *μ*g/mL), phosphate buffer (2.5 mL, 0.2 M, pH 6.6), and potassium ferricyanide (1 mL, 1%, *w*/*v*) were incubated (50°C, 20 min). Whereafter, trichloroacetic acid (2 mL, 10%, *w*/*v*) and ferric chloride (1.2 mL, 0.1%, *w*/*v*) were added for terminating the above reaction. Finally, OD_700 nm_ was determined. During the antioxidant analysis *in vitro*, deionized water and vitamin C (Vc) were used as a blank and a positive control, respectively.

The scavenging activity towards DPPH radical was measured according to the method reported by Zhao et al. [25]. The mixture including AE-PS (0.2 mL, 0–400 *μ*g/mL) and DPPH solution (0.6 mL, 0.004%, *w*/*v* in methanol) was disposed at the dark and still standing for 30 min. Subsequently, OD_517 nm_ was measured and the scavenging rate was calculated on the basis of the following formula:

(1) $$\begin{array}{c} Scavenging\;rate\left(\%\right)=\left[\frac{A_{0}–A_{1}}{A_{1}}\right]\times 100, \end{array}$$

where *A*_0_ is the absorbance of the blank and *A*_1_ is the absorbance of AE-PS or Vc.

The scavenging hydroxyl radical ability was evaluated by the previously reported method [26]. Ferrous sulfate (1 mL, 9 mM), the AE-PS (1 mL, 0–400 *μ*g/mL), salicylic acid (1 mL, 9 mM), and hydrogen peroxide (1 mL, 8.8 mM) were kept for 30 min at 37°C and centrifuged (3000 rpm, 10 min). OD_510 nm_ was determined, and the scavenging rate was calculated by the following formula:

(2) $$\begin{array}{c} Scavenging\;rate\left(\%\right)=\left[\frac{A_{0}–A_{1}}{A_{0}}\right]\times 100, \end{array}$$

where *A*_0_ is the absorbance of the blank and *A*_1_ is the absorbance of AE-PS or Vc.

The IC_50_ values (*μ*g/mL) of scavenging DPPH or hydroxyl radical were defined as the effective concentrations of the sample at which the radicals were inhibited by 50%.

### 2.4. Acute Toxicity Study

The experiments were performed according to procedures approved by the Institutional Animal Care and Use Committee of Shandong Agricultural University in accordance with the Animals (Scientific Procedures) Act 1986 (amended 2013). Acute toxicity test of mice complied with a previously reported method [27]. Twenty mice were averagely divided into one AE-PS-treated group and one normal saline group. The AE-PS-treated group was treated with 5000 mg AE-PS/kg, and the normal saline group was administered with normal saline of the same volume. During this experiment, all mice had free access to food and water and were observed for mortality and behavioural changes for 14 days.

### 2.5. Animal Experiment

The seventy mice were maintained in a 12 h light/dark cycle, temperature 23 ± 2°C, humidity 50 ± 5% animal room with free access to food and water. After a week of domestication, six mice were fed the normal diets as the normal control (NC) group for the diabetic group. The other mice as the diabetic group were fed HFD (15% lard, 2% cholesterol, 0.3% sodium cholate, 0.7% salt, 5% white sugar, and 77% regular diet). After 4 weeks, the mice in the diabetic group were fasted for 12 h but with free access to water and injected intraperitoneally twice with 60 mg/kg STZ solution in a citrate buffer within 72 h, while the NC group mice were injected intraperitoneally with isometric physiological saline. After feeding for 3 d, blood glucose levels of the diabetic mice were evaluated by taking a drop of blood from the tip of the tail with a glucometer (Sano Bio-sensing Technology Co. Ltd., Changsha, China). Mice with blood glucose concentrations over 11.1 mM were identified as the diabetic mice and chosen for further pharmacological studies.

Twenty-four diabetic mice were selected with the closest body weight and blood glucose level and then randomized into four groups of 6 mice each including one model control (MC) group, one glimepiride (GL) group, and two AE-PS-treated groups. In the GL group, mice were treated with glimepiride (2 mg/kg). In the AE-PS-treated groups, mice were administered with high level (HAE-PS, 400 mg/kg) and low level (LAE-PS, 100 mg/kg). In the NC and the MC groups, mice received saline of the same volume. Each treatment lasted for 4 weeks, and all mice had free access to food and water.

During the experiment, body weights and fasting blood glucose (FBG) of all mice were recorded for three times at 0, 4, and 8 weeks, respectively. At the last week of treatment, oral glucose tolerance test was also performed according to the previous method [28]. The overnight-fasted mice were treated with 2 g/kg glucose solution by oral gavage, and blood samples were obtained from the tail vein to evaluate the blood glucose levels at 0, 30, 60, 90, and 120 min.

All mice were fasted for one night at the end of the experiment and then sacrificed quickly by euthanasia. Blood samples were collected from the orbital sinus and centrifuged (4000 rpm, 10 min, 4°C) to obtain the serum, and the liver, kidney, and pancreas were surgically removed, weighed, and homogenized immediately in phosphate buffer solutions (0.2 M, pH 7.4) and centrifuged (3000 rpm, 10 min, 4°C) to offer homogenates for further biochemical assay. The serum total cholesterol (TC), triglyceride (TG), low-density lipoprotein cholesterol (LDL-C), high-density lipoprotein cholesterol (HDL-C), alanine aminotransferase (ALT), aspartate aminotransferase (AST), urea nitrogen (BUN), and creatinine (CRE) were evaluated using an automatic biochemical analyzer (BS-380, Shenzhen, China). The SOD, GSH-Px, CAT, and MDA in the liver, kidney, pancreas, and blood as well as insulin of serum were evaluated using commercial reagent kits by manufacturer's instructions. The slices of the liver, kidney, and pancreas were prepared for histopathological observations by the method of a previously published study [25].

### 2.6. Gas Chromatography (GC), Fourier-Transform Infrared (FTIR), and Nuclear Magnetic Resonance (NMR) Spectroscopy Analysis

The monosaccharide composition of AE-PS was calculated by a GC (GC-2010, Shimadzu, Japan) equipped with a hydrogen flame ionization detector. The AE-PS and standard monosaccharides (xylose, ribose, arabinose, mannose, galactose, glucose, rhamnose, and fucose) were prepared according to our published method [29]. The processed supernate (1 *μ*L) was injected into an HP-5 fused silica capillary column (3000 × 0.32 × 0.25 mm) and analyzed by the standard curves of standard monosaccharides.

The FT-IR spectrum of AE-PS was analyzed using an infrared spectrometer (Nicolet 6700, Thermo Fisher Scientific, USA) in the frequency range of 4000–500 cm^−1^ by the potassium bromide disc method.

The NMR spectrum of AE-PS in deuterated water was recorded by a Bruker AV-300 spectrometer operating at 300 MHz at 25°C.

### 2.7. Statistical Analysis

Statistical analyses were performed by SPSS software. All data were expressed as the means ± SD (standard deviations). Differences among experimental groups were known as statistically significant if *P* < 0.05 by one-way ANOVA of Duncan's multiple range tests.

## 3. Results

### 3.1. In Vitro Antioxidant Abilities of AE-PS

**Figure 1:**
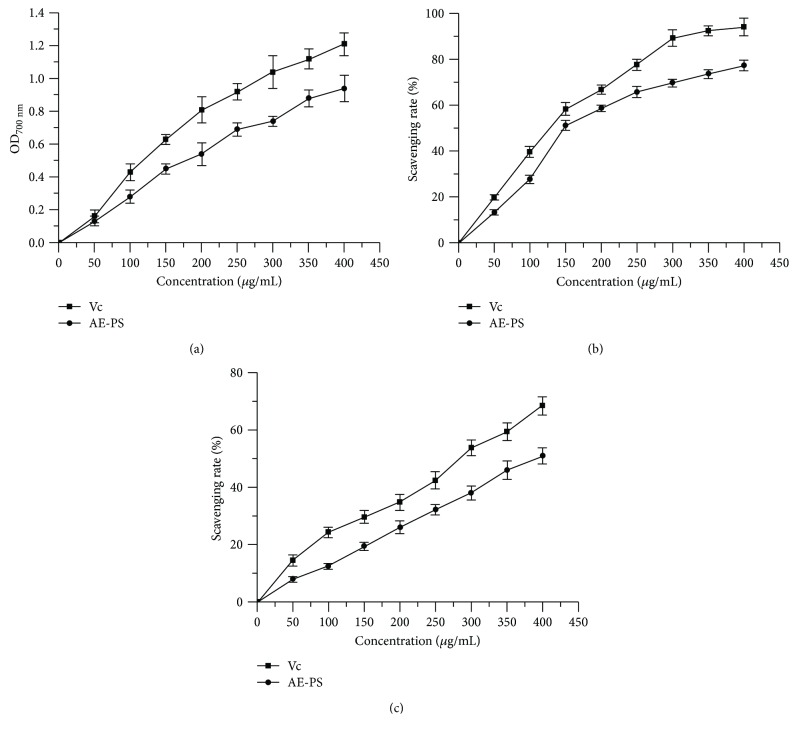
Antioxidant activities of AE-PS *in vitro*: (a) reducing power, (b) scavenging rate towards DPPH, and (c) scavenging rate towards hydroxyl radical.

The higher absorbance value of the sample is, the stronger its reducing power is. The reducing powers of AE-PS and Vc are depicted in Figure 1(a). The reducing power of AE-PS was elevated with increasing sample concentration from 0 to 400 *μ*g/mL. At 400 *μ*g/mL, the reducing power of AE-PS reached 0.94 ± 0.08.

The scavenging activity of samples toward DPPH radical was reflected by original color changes of the liquid. The scavenging ability of AE-PS on DPPH radical was in a concentration-dependent manner (Figure 1(b)). At 400 *μ*g/mL, the scavenging abilities of Vc and AE-PS on DPPH radical were 94.19 ± 3.91% and 77.28 ± 2.24% and the IC_50_ values of Vc and AE-PS reached 119.475 ± 2.077 *μ*g/mL and 167.125 ± 2.223 *μ*g/mL, respectively.

Obviously, the scavenging activities of Vc and AE-PS on hydroxyl radicals were well positively correlated with the concentrations (Figure 1(c)). The scavenging activity of Vc and AE-PS reached 68.41 ± 3.20 and 50.91 ± 2.87% at the concentration of 400 *μ*g/mL, and the IC_50_ values of Vc and AE-PS were 271.709 ± 2.434 and 419.720 ± 2.623 *μ*g/mL.

### 3.2. Acute Toxicity Study

The abnormal behaviours and deaths were not observed in the tested mice by the acute toxicity test, indicating AE-PS was practically a nontoxic substance.

### 3.3. Effects of AE-PS on Body Weights and Organ Indexes

**Figure 2:**
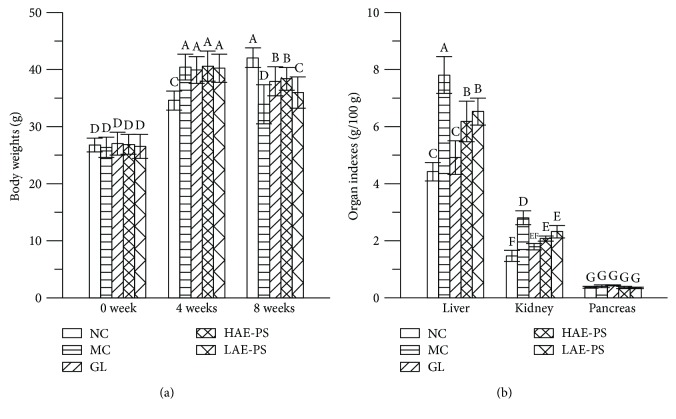
Effect of AE-PS on body weights and organ indexes in T2DM mice. (a) Body weights and (b) organ indexes. The values are reported as the means ± SD. Bars with different letters are significantly different (*P* < 0.05).

The body weights and organ indexes of all experimental mice in three different experimental stages are demonstrated in Figure 2. The initial body weights of mice among all groups have no significant differences. After the modeling process for 4 weeks, the body weight in the NC group was obviously lower than those in other groups (*P* < 0.05). After 8 weeks, the MC group expressed a significant decrease in body weight and a distinct increase in liver and kidney indexes compared to the NC group (*p* < 0.05), while oral administration with AE-PS and GL obviously increased the body weights and decreased the liver and kidney indexes when compared with those of the MC group (*P* < 0.05). However, there were no statistically significant differences in pancreas index among all groups.

### 3.4. Effects of AE-PS on FBG, Serum Insulin Levels, and Oral Glucose Tolerance Ability

**Figure 3:**
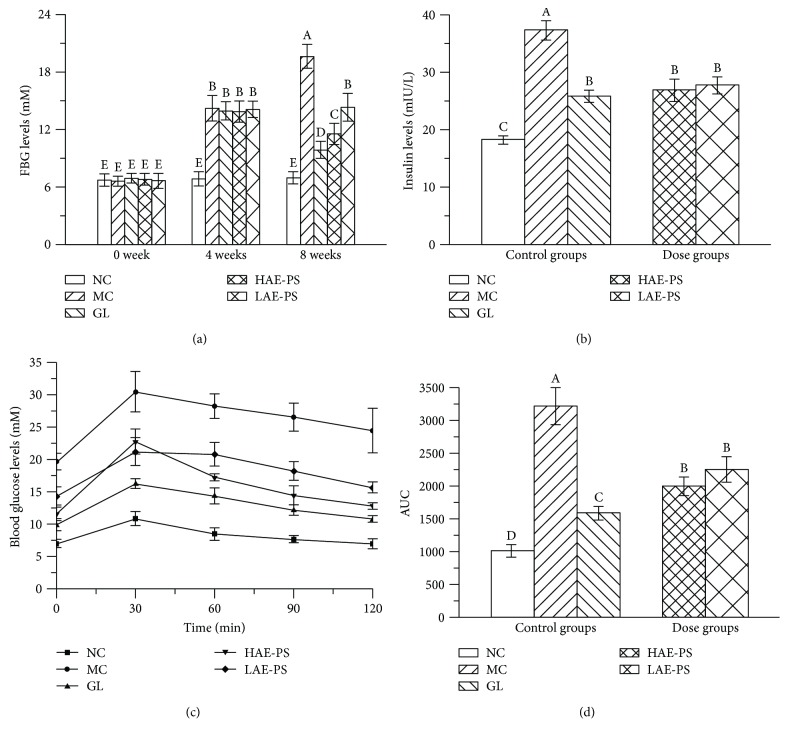
Effects of AE-PS on FBG, serum insulin levels, and oral glucose tolerance ability in T2DM mice. (a) FBG levels, (b) insulin levels, and oral glucose tolerance test: (c) blood glucose levels and (d) AUC. The values are reported as the means ± SD. Bars with different letters are significantly different (*P* < 0.05).

The FBG levels in three different experimental stages are summarized in Figure 3(a). At 0 week, FBG levels among the groups had no marked changes. At 4 weeks, FBG level of mice in the NC group was lower than those of the MC, GL, and dose groups (*P* < 0.05) and FBG levels of the MC, GL, and dose groups were over 11.1 mM, indicating the T2DM model was successfully established. At the end of the experiment, the MC group showed observable elevation in FBG level compared to the NC group (*P* < 0.05). However, treatment with different doses of AE-PS or GL for four weeks exhibited noticeable reduction when compared with the MC group (*P* < 0.05).

As exhibited in Figure 3(b), the serum insulin level in the MC group displayed marked upgrade compared with that in the NC group (*P* < 0.05), showing that insulin resistance had occurred in diabetic mice. Besides, serum insulin levels in the GL and AE-PS groups were decreased when compared to that in the MC group.

The change of each group in the blood glucose level after oral administration of glucose (2 g/kg) is shown in Figure 3(c). The blood glucose levels of all experimental groups reached the peak at 30 min, and then the NC group was gradually restored to the initial level at 120 min. However, the blood glucose level in other groups had been invariably kept at a high level during the whole test. Furthermore, the area under the curve (AUC) was calculated by GraphPad Prism 5 (Figure 3(d)). AUC (3217.0 ± 285.0) in the MC group was higher than that (1014.0 ± 100.2) in the NC group (*P* < 0.05). Administration of AE-PS and GL gave rise to a remarkable suppression on the AUC compared with the MC group.

### 3.5. Effect of AE-PS on AST, ALT, BUN, and CRE

**Figure 4:**
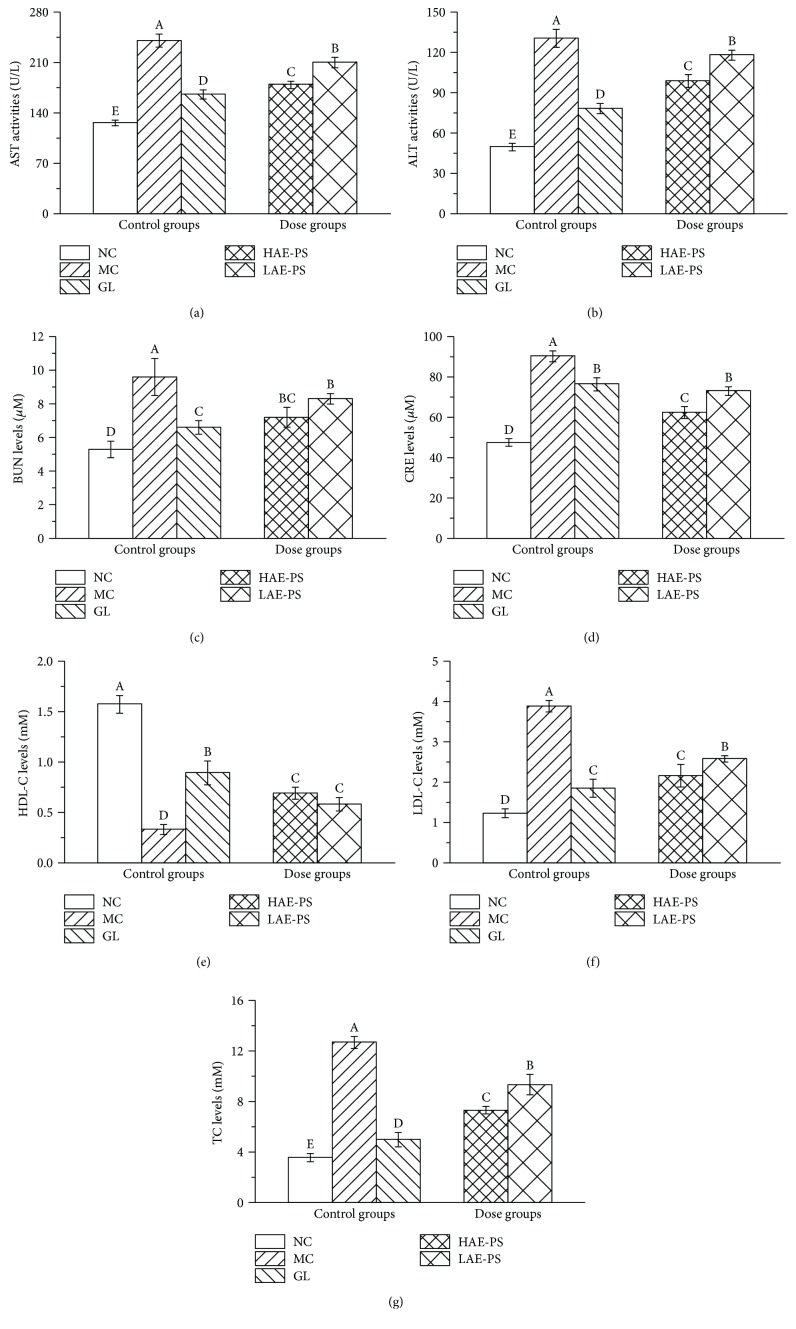
Effect of AE-PS on serum properties in T2DM mice: (a) AST, (b) ALT, (c) BUN, (d) CRE, (e) HDL-C, (f) LDL-C, and (g) TC. The values are reported as the means ± SD. Bars with different letters are significantly different (*P* < 0.05).

As demonstrated in Figures 4(a)–4(d), the AST, ALT activities and BUN, and CRE levels showed a remarkable rise in the MC group when compared with those in the NC group (all with *P* < 0.05), indicating that the liver and kidney were damaged. By contrast, pretreatment with AE-PS markedly inhibited the abnormal increases among these indexes (*P* < 0.05) compared to those in the MC group.

### 3.6. Effect of AE-PS on Lipid Metabolic Parameters

The LDL-C and TC levels increased, whereas HDL-C level decreased in the MC group when compared to that in the NC group (*P* < 0.05, Figures 4(e)–4(g)). Interestingly, the abnormal increase of LDL-C and TC levels and the decrease of HDL-C level were significantly ameliorated by supplementation of AE-PS compared with those in the MC group.

### 3.7. Histopathological Study of the Liver and Kidney

**Figure 5:**
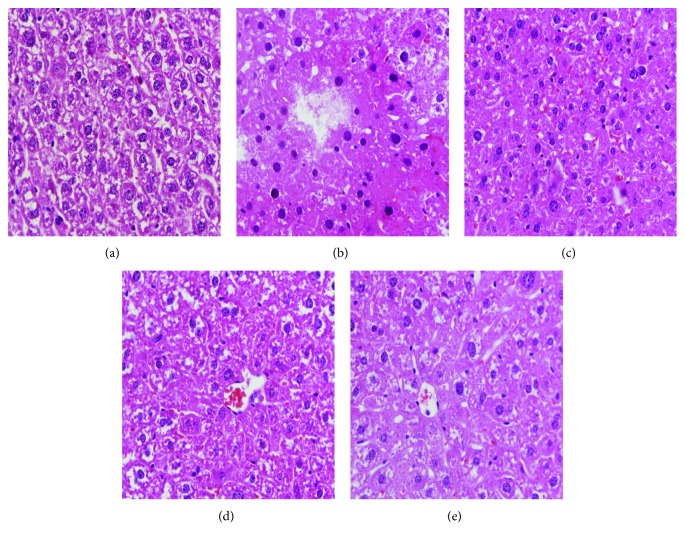
Optical micrographs of mouse liver sections (400 × magnification) in T2DM mice. (a) NC group, (b) MC group, (c) GL group, (d) HAE-PS group, and (e) LAE-PS group.

**Figure 6:**
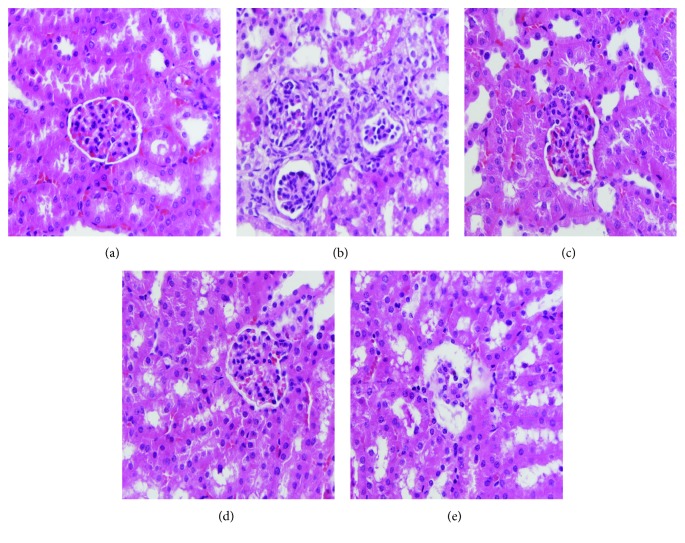
Optical micrographs of mouse kidney sections (400 × magnification) in T2DM mice. (a) NC group, (b) MC group, (c) GL group, (d) HAE-PS group, and (e) LAE-PS group.

Images of hepatic and renal histological sections are demonstrated in Figures 5 and 6. Obvious liver damage, characterized by cellular degeneration and hepatocyte necrosis, was found in the MC group when compared to that in the NC group (Figures 5(a) and 5(b)). Glomerulus destruction, glomerular sclerosis, vacuolation of tubular epithelial cells, and loss of brush border were observed in the kidney section of the MC group compared with that of the NC group (Figures 6(a) and 6(b)). The liver and kidney sections of the mice treated with AE-PS (100 and 400 mg/kg) obviously improved these injuries in comparison with that in the MC group (Figures 5(d), 5(e), 6(d), and 6(e)).

### 3.8. Effect of AE-PS on Antioxidant Enzymes and Lipid Peroxide

**Figure 7:**
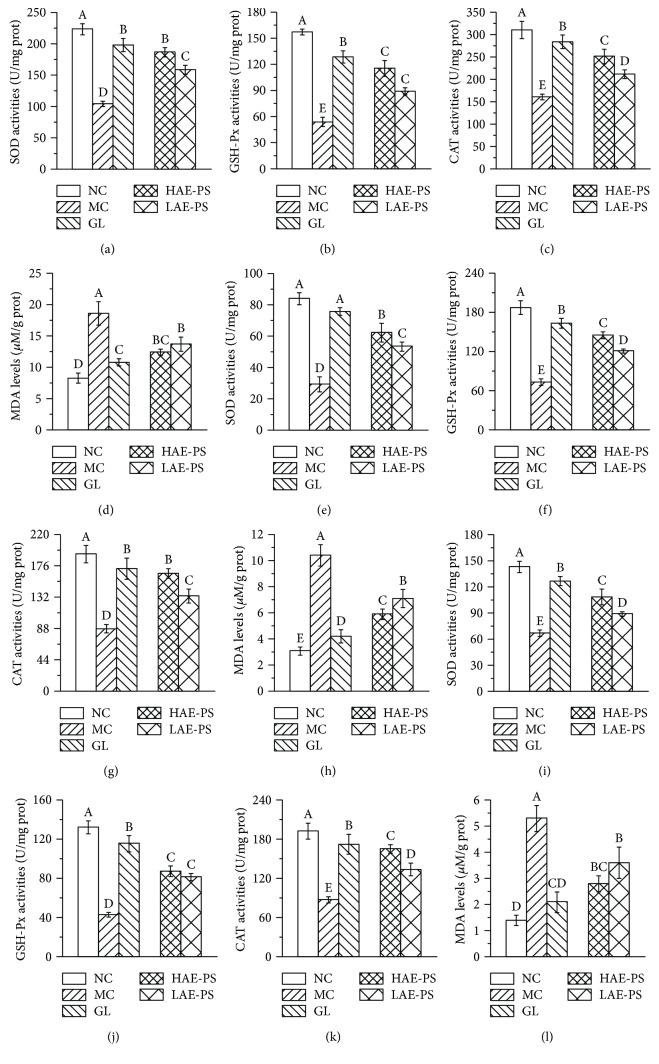
Effect of AE-PS on SOD, GSH-Px, CAT, and MDA in T2DM mice. (a–d) in hepatic homogenates, (e–h) in renal homogenates, and (i–l) in pancreatic homogenates, respectively. The values are reported as the means ± SD. Bars with different letters are significantly different (*P* < 0.05).

As shown in Figures 7(a)–7(c), 7(e)–7(g), and 7(i)–7(k), the significant decreases in hepatic, renal, and pancreatic SOD, GSH-Px, and CAT activities were found in HFD- and STZ-induced diabetic mice when compared with those in the NC group (*P* < 0.05), demonstrating that oxidative stress had occurred in diabetic mice. However, treatment with AE-PS elevated these parameters as compared to those in the MC group. The activities of hepatic SOD, GSH-Px, and CAT increased by 79.2%, 113.7%, and 56.8% in the HAE-PS group, as well as 51.8%, 64.3%, and 31.4% in the LAE-PS group, respectively, compared to those of the MC group. A similar tendency of AE-PS on SOD, CAT, and CAT activities was observed in the homogenates of the kidneys and pancreas; the SOD, GSH-Px, and CAT activities in the HAE-PS group were 119.0%, 97.1%, and 89.1% in the kidney, as well as 61.3%, 102.1%, and 156.9% in the pancreas higher than those in the MC group.

Furthermore, lipid peroxidation such as MDA was also assessed (Figures 7(d), 7(h), and 7(l)). The MDA level of the MC group was higher than that of the NC group (*P* < 0.05) in the livers, kidneys, and pancreas. After the supplement of AE-PS, the increased tendencies were significantly attenuated.

### 3.9. GC, FT-IR, and NMR Spectroscopy Analysis

**Figure 8:**
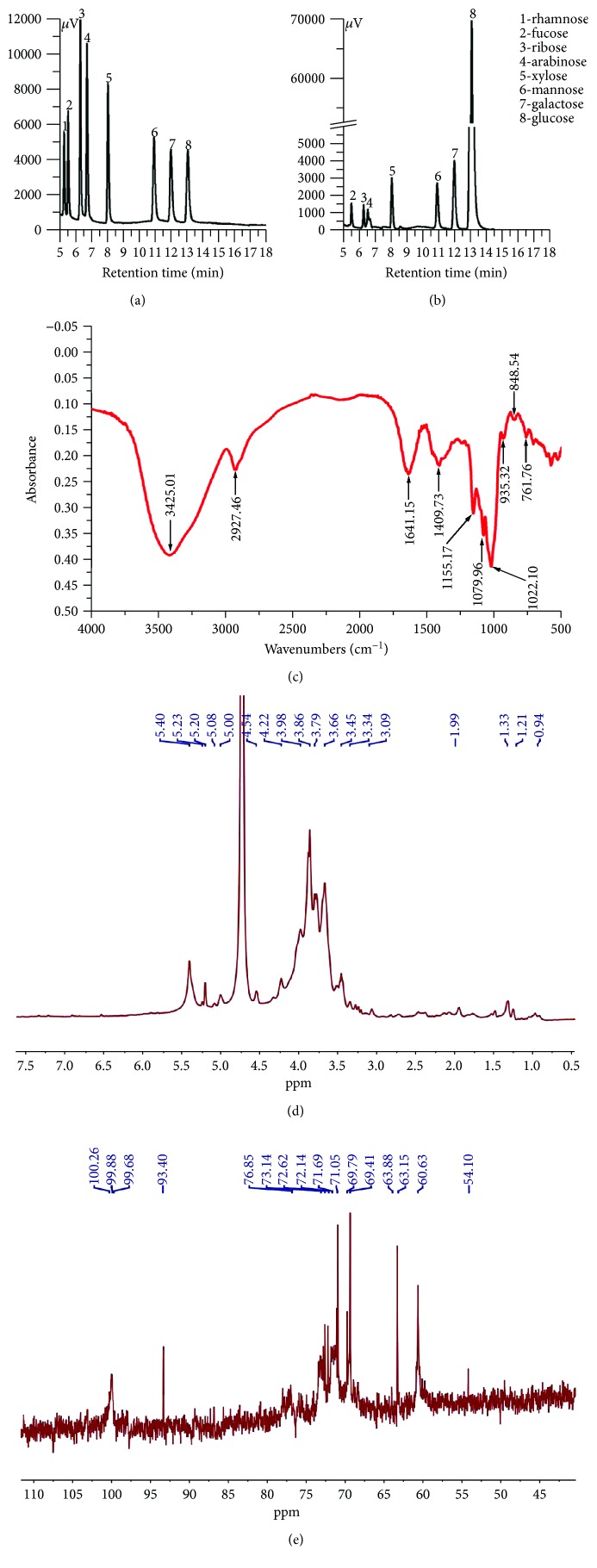
Preliminary characterizations: (a) GC chromatograms of standard monosaccharides, (b) GC chromatograms of AE-PS, (c) FT-IR, (d) ^1^H NMR, and (e) ^13^C NMR.

The monosaccharide composition of AE-PS was measured by comparison with the retention times and chromatograph peaks of standard monosaccharides including xylose, ribose, arabinose, mannose, galactose, glucose, rhamnose, and fucose (Figure 8(a)). The AE-PS was made up of seven different monosaccharides of fucose, ribose, arabinose, xylose, mannose, galactose, and glucose with mass percentages of 1.23%, 0.57%, 0.29%, 2.12%, 2.73%, 4.66%, and 88.4%, respectively (Figure 8(b)), showing that AE-PS was heterogeneous and the glucose is the major component.

The FT-IR spectra were powerful techniques, which can well identify characteristic functional and organic groups. A wide and strong band at 3425.01 cm^−1^ of –OH stretching vibrations, a weak peak at 2927.46 cm^−1^ of C–H bending vibrations, a peak at 1641.15 cm^−1^ of C=O asymmetric vibrations, and a peak at 1409.3 cm^−1^ of –COOH stretching vibrations are displayed in Figure 8(c) in the FT-IR spectra of AE-PS at the range of 500–4000 cm^−1^. Furthermore, a strong extensive absorption at 1200–900 cm^−1^ for coupled C–O–C glycosidic band vibrations and C–O–H bending vibrations of side groups indicated the characteristic absorptions of polysaccharides [30]. The diagnostic absorption peaks at 915.31 cm^−1^, 848.54 cm^−1^, and 761.76 cm^−1^ may suggest the presence of *β*-D-pyranoid glucose and *α*-isomers of pyranose [31]. Based on the results mentioned above, it can draw a conclusion that AE-PS was a pyranose form sugar with *α*- and *β*-configurations.

As shown in Figure 8(d), the signals of AE-PS were distributed at *δ*_H_ 3.0–5.4, which were the typical NMR signals of the polysaccharides [32]. The chemical shifts of anomeric protons at 5.40 ppm, 5.23 ppm, 5.20 ppm, 5.08 ppm, 5.00 ppm, 4.54 ppm, and 4.22 ppm indicated the existence of both *α*- and *β*-configurations in AE-PS [33]. The signals from 3.09 ppm to 3.98 ppm were attributed to atoms H2–H6. The multitudes of signals at *δ*_H_ 0.94–1.99 were known as the attributions of the N–CH_3_ and N–H groups. In ^13^C NMR spectrum (Figure 8(e)), the signals at 100.26 ppm, 99.88 ppm, 99.68 ppm, and 93.40 ppm manifested both *α*- and *β*-anomeric configurations existing in AE-PS [34]. The signals at *δ*_H_ 76.85–60.63 were attributed to C2–C6. Furthermore, the signals at 5.40 ppm, 5.23 ppm, 5.20 ppm, 5.08 ppm, and 5.00 ppm in ^1^H NMR spectrum were assigned to anomeric protons of *α*-glucopyranose units in the main chain, which was supported by the signal at 93.40 ppm in ^13^C NMR spectrum [35, 36]. The chemical shift of anomeric proton at 4.54 ppm and 4.22 ppm and the ^13^C chemical shift at 100.26 ppm, 99.88 ppm, and 99.68 ppm could be assigned to *β*-galactopyranose [37, 38]. The results of NMR analysis were also confirmed by the analysis of GC and FT-IR.

## 4. Discussion

The etiology of T2DM mainly reflects in defects of insulin secretion and insulin resistance development. The abnormal insulin secretion is due to the functional deficit and loss of pancreatic *β*-cells, which can primarily cause hyperglycemia and subsequently reduce the sensitivity of insulin [39, 40]. Therefore, an experimental animal model should imitate pathogenesis and clinical features of individuals with T2DM. At present, animal models, widely used in experimental studies, contain KK/A^y^, db/db, and ob/ob mice, which possess inherited hyperglycemia and insulin resistance [41]. Nevertheless, these animal models are more expensive, more difficult to breed, kept constant at pathological conditions, and unsuitable for studies of insulin secretagogues because of serious insulin resistance. Many literatures have reported that the T2DM model, established by feeding mice HFD for causing insulin resistance and then injecting STZ, is comparatively cheap and easier and closely reflects the pathological features of T2DM [1, 39]. In our work, mice were fed HFD for 4 weeks and then injected intraperitoneally twice with 60 mg/kg STZ solution in a citrate buffer within 72 h to establish the T2DM model.

Body weight loss, a common feature of diabetes, may be due to inefficient utilization of most proteins and carbohydrates [42]. In this work, the diabetic mice induced by HFD and STZ exhibited a remarkable decline in body weight as well as a remarkable increase in organ indexes of the liver and kidney, which was in line with the results reported by Li et al. [43]. However, the loss of body weight and increase of organ indexes were notably improved by the administration with AE-PS. Compared with the polysaccharides of *Inonotus obliquus* at the dosage of 900 mg/kg, the growth rate of body weight (13.03%), and the decline rate of liver index (20.87%) and kidney index (25.98%) of mice in the HAE-PS group were higher than those of mice treated with *I. obliquus* polysaccharides [1]. These results indicated that AE-PS possessed a good therapeutic effect on T2DM-induced body weight loss and organ indexes increase.

FBG and oral glucose tolerance ability tests are the two most common tests for the diagnosis of diabetes [44]. Hence, both FBG and oral glucose tolerance ability were investigated in the present study during the treatment. In our work, the injection of STZ obviously increased the FBG levels and deteriorated the oral glucose tolerance ability. However, it was obvious that the supplement of AE-PS could decrease FBG concentrations and improve deteriorated oral glucose tolerance ability in T2DM mice, especially AE-PS at the dose of 400 mg/kg.

Furthermore, the insulin resistance is commonly used as an index for assessing diabetes. The feedback loop of insulin is the simplest mechanism under the foundation state, and the interaction of *β* cells, liver, and peripheral tissues maintains the dynamic equilibrium of insulin levels [45]. The strict control of insulin plays an important role in preventing and treating diabetes. In our research, the T2DM significantly enhanced insulin level of the diabetic groups, whereas AE-PS treatment reduced the insulin level, manifesting that AE-PS could mitigate the insulin resistance thereby achieving a therapeutic effect on T2DM.

Many reports have manifested that T2DM is closely in connection with dyslipidemia due to the decrease in sensitivity of fat cell membrane receptors to insulin, leading the weakening of antifat effect and causing the accumulation of fatty acids in the blood [45, 46]. From our work, the administration of AE-PS significantly decreased TC and LDL-C levels and remarkably increased HDL-C levels in diabetic mice, indicating that AE-PS could have positive effects on hyperlipidemia induced by T2DM to lower risk of cardiovascular disease.

Clinically, the serums AST and ALT are commonly used as biochemical markers for assessing liver damage, and their activities are observably elevated when liver injury occurred, which is due to damaged permeability of hepatocytic membrane which causes ALT and AST permeated into blood circulation [25]. The physical status of the kidney is diagnosed by monitoring dynamic changes of BUN and CRE levels [22]. The BUN, an endogenous substance, is generated by hepatic protein decomposition and excreted *via* glomerulus filtration. The CRE is an endogenous by-product of creatine and phosphocreatine catabolism and then discharged to body fluids. The present work showed that AE-PS could repair the damages of hepatic and nephritic tissues in the diabetic mice. Furthermore, the protective effects on the liver and kidney could be confirmed by the histopathological examinations clinically. Presently, the H&E staining on the liver and kidney in T2DM mice was also processed to verify the protection at the cellular level. The results indicated that the STZ-induced liver and kidney damage could be inhibited by AE-PS, which was also confirmed by the results of AST, ALT, BUN, and CRE evaluations.

Diabetes is related to oxidative stress induced by an imbalance between antioxidant ability and levels of free radicals and/or reactive oxygen in organisms, which is due to insulin resistance which can reduce the utilization of glucose and augment the levels of oxygen-derived free radicals [45]. To well estimate the antidiabetic ability of AE-PS, *in vitro* and *in vivo*, its antioxidant abilities were analyzed. Reducing power is commonly concerned with the existence of reductones, which can play to give antioxidant action by donating a hydrogen atom when the free radical chain is broke [25]. DPPH as a stable free radical compound has been a wide indicator and a quick method for testing the scavenging ability of various antioxidative samples towards free radical [21]. Hydroxyl radical, the strongest chemical activity among free radicals and/or reactive oxygen, can destroy many biomolecules such as protein, RNA, and DNA [17]. Based on these results, AE-PS showed potential for development as a natural antioxidant for preventing and treating ROS-induced diseases. Furthermore, Jing et al. [21] have investigated the antioxidant ability of water-extractable polysaccharides. Its reducing property (0.62) was lower than that (0.94) of AE-PS at 400 *μ*g/mL, and its IC_50_ values to scavenge DPPH (1150 *μ*g/mL) and hydroxyl radical (548 *μ*g/mL) were higher compared to those of AE-PS, indicating that AE-PS had a stronger antioxidant ability. The antioxidant enzymes *in vivo* including SOD, GSH-Px, and CAT could defend against ROS formation under the oxidative stress, with the possible mechanism that SOD can activate the dismutation of superoxide radicals to hydrogen peroxide, which is finally decomposed into H_2_O_2_ and O_2_ by GSH-Px and CAT, resulting in the prevention of ROS formation [29, 47, 48]. In our study, AE-PS inhibited a decrease in SOD, GSH-Px, and CAT of the liver, kidneys, and pancreas in the HFD- and STZ-induced diabetic mice. The results testified that AE-PS had potentially been treated and prevented against T2DM by elevating antioxidant effects *in vivo*. Furthermore, free radicals and/or reactive oxygen could interact with polyunsaturated fatty acids to form lipid peroxidation (MDA), which is deemed to be a marker of oxidative stress-induced tissue injuries [49]. However, the abnormal MDA levels in the livers, kidneys, and pancreas of the HFD and STZ-induced diabetic mice were inhibited by the administration of AE-PS, indicating that AE-PS could eliminate free radicals and/or reactive oxygen-induced lipid peroxidation.

GC evaluation found that AE-PS was made up of fucose, ribose, arabinose, xylose, mannose, galactose, and glucose, which was different from CMP-W1 (mannose, galactose, and glucose) by subcritical water extraction, CBP-1 (mannose, galactose, and glucose) by alkaline extraction from fruiting bodies, and acidic polysaccharides (xylose, arabinose, galactose and rhamnose) from mycelium of *C. militaris* [50]. Furthermore, AE-PS was an *α*- and *β*-configuration polysaccharide by FT-IR and NMR, which was different from CSP and SeCSP-II (*β*-configuration) of *C. militaris* [51]. These different results may be associated with extraction conditions and sample states. Moreover, the monosaccharide composition and structure of mushroom polysaccharide may play important roles in its biological activities [17, 52, 53]. Lu et al. have reported that the *Auricularia auricular* polysaccharides possess a remarkable hypoglycemic effect in diabetic mice [54]. Zhao et al. [25], Song et al. [55], and Ma et al. [56] have reported that fucose plays an important role in conferring higher biological activities, and the *β*-type glycosidic linkages could maintain the biological activities. AE-PS exhibited antioxidant and hypoglycemic activities, as well as protective effects on the liver and kidney, which may be related to the properties of that *β*-type glycosidic linkages and heteropolysaccharide containing fucose.

## 5. Conclusions

This work showed that the AE-PS from *C. militaris* was possessed of potential antioxidant, antihyperlipidemic, and hypoglycemic activities, improved insulin resistance, and protected the liver, kidneys, and pancreas in the T2DM mice, suggesting that AE-PS could be used as a potentially natural nontoxic and functional food for the prevention and treatment of T2DM and its complications induced by HFD and STZ. Furthermore, GC analysis exerted that AE-PS was heterogeneous and glucose was the major monosaccharide component. FT-IR and NMR analysis indicated that AE-PS was a pyran-ring polysaccharide with *α*- and *β*-configurations.

## Data Availability

The data used to support the findings of this study are available from the corresponding author upon request.

## Abbreviations

AE-PS: Acidic-extractable polysaccharides
ALT: Alanine aminotransferase
AST: Aspartate aminotransferase
AUC: Area under the curve
BUN: Urea nitrogen
CAT: Catalase
CRE: Creatinine
DPPH: 1,1-Diphenyl-2-picrylhydrazyl
DM: Diabetes mellitus
FBG: Fasting blood glucose
FTIR: Fourier-transform infrared
GC: Gas chromatography
GL: Glimepiride
GSH-Px: Glutathione peroxidase
HDL-C: High-density lipoprotein cholesterol
HFD: High-fat diet
LDL-C: Low-density lipoprotein cholesterol
MC: Model control
MDA: Malondialdehyde
NC: Normal control
NMR: Nuclear magnetic resonance
SD: Standard deviations
SOD: Superoxide dismutase
STZ: Streptozotocin
T2DM: Type 2 diabetes mellitus
TC: Total cholesterol
TG: Triglyceride
Vc: Vitamin C.
